# Highlighting the genetics of natural and synthetic selfish elements in a special GENETICS and G3 series

**DOI:** 10.1093/genetics/iyag160

**Published:** 2026-08-05

**Authors:** Amanda M Larracuente, Fred Gould, Kelly A Dyer

**Affiliations:** Department of Biology, University of Rochester, Rochester, NY, United States; Department of Entomology and Plant Pathology, North Carolina State University, Raleigh, NC, United States; Department of Genetics, University of Georgia, Athens, GA, United States

Selfish elements bias their own transmission to the next generation, often at an expense to the host that harbors them (reviewed in Werren et al. 1988; Burt and Trivers 2008). Once considered oddities, it is now clear that selfish elements take many forms and are widespread across the tree of life. Early studies laid the foundation for the presence and evolutionary dynamics of selfish elements, and modern investigations show their far-reaching consequences for molecular processes and genome evolution (reviewed in Presgraves et al. 2026). In complement, based on an understanding of naturally occurring selfish element mechanisms and dynamics, synthetic genetic elements are being developed to suppress or alter traits of pest species.

Recognizing the broad importance of these selfish elements, the GSA journals have established a series to highlight the genetic mechanisms and evolutionary consequences of selfish non-Mendelian inheritance. We invited high-quality submissions with a focus on selfish non-Mendelian transmission, including both theoretical and empirical studies of natural and synthetic systems. This first issue of the series features natural selfish genetic elements—including genes, chromosomes, endosymbiotic bacteria, and autonomous plasmids—and synthetic selfish genetic elements (SSGEs). Together, these studies investigate the dynamics, mechanisms, genomic features, and consequences of selfish elements.

## Natural selfish genetic element systems

Meiotic drivers violate Mendelian principles by cheating meiosis, biasing their segregation to the next generation. The first detailed investigation of sex chromosome meiotic drive was published nearly 100 years ago in GENETICS (Gershenson 1928). Since that landmark paper, many empirical studies in plants, animals, and fungi have revealed the myriad ways selfish genetic elements can distort segregation ratios and bias their transmission to the next generation. The GSA journals have been the home for much of the foundational and recent work on non-Mendelian segregation (Edvalson and Larracuente 2025).

Meiotic drive is well-documented in *Drosophila* species, and most of these systems are sperm killers that cause sex ratio distortion (Jaenike 2001). The *Stellate* system of *Drosophila melanogaster* has long been viewed as a relic sex ratio drive system whose effects are largely suppressed in contemporary populations. In this issue, McCormick and Clark challenge this view, using genetics to demonstrate that *Stellate* likely behaves as an active driver in natural populations (McCormick et al. 2026). Complementing this population-level perspective, Meng and Yamashita uncover a mechanism involved in *Stellate*-mediated sperm dysfunction. They show that *Stellate* induces defects in nuclear envelope remodeling during spermatid development, revealing one way that meiotic drivers can exploit spermatogenesis to bias their transmission (Meng and Yamashita 2026).

Despite their ability to gain a transmission advantage, some meiotic drivers are maintained at low population frequencies, raising questions about factors that limit their spread. One possible explanation is that meiotic drive usually comes at a cost, prompting the evolution of resistance or suppression. Chang et al. revisit this question using the classic *Segregation Distorter* system in *D. melanogaster* (Chang et al. 2026). Using genomic and genetic analyses, they disentangle resistance at the target locus (a highly repetitive satellite DNA) from unlinked suppressors elsewhere in the genome to show that widespread suppressors are a major force limiting the spread of *Segregation Distorter* in a natural population.

Female meiosis presents an opportunity for meiotic drive due to the asymmetry in the outcomes of meiosis. Centromeres can behave selfishly and bias their inheritance through female meiosis. The factors contributing to female meiotic drive are not well understood. In this issue, Gorringe et al. study the effects of natural genetic variation on recombination rates near centromeres and segregation distortion in *Arabidopsis thaliana* (Gorringe et al. 2026). The authors measure crossover rates and marker transmission in crosses between diverse Arabidopsis accessions, using fluorescent markers flanking the centromeres. They observe segregation distortion in some hybrid combinations, suggesting that *cis* and *trans* genetic factors, and their interactions, affect variation in recombination and segregation distortion (Gorringe et al. 2026). This work adds new insights into ways that structural variation in centromeres can influence recombination and segregation distortion.

While meiotic drive is well-documented in model organisms, evidence in humans is limited. In this issue, Dai and Costain systematically review reports of transmission ratio distortion in humans and find that most claims are preliminary or conflicting and confounded by limited dataset sizes and ascertainment bias (Dai and Costain 2026). With the growing availability of large-scale human genomic data, revisiting this question is timely. For now, whether true transmission distortion exists in humans remains an open question.

Some selfish elements are extra-chromosomal, meaning they are not an essential component of the genome. For instance, B chromosomes are supernumerary chromosomes that ensure their transmission despite not being essential for development (Ferree et al. 2024). In this issue, Ferretti et al. characterize the genomic and epigenetic landscape of a B chromosome in *D. melanogaster* (Ferretti et al. 2026). Their analyses reveal a repeat-rich chromosome landscape with satellite DNAs, transposable elements, and heterochromatic marks, providing new insight into the composition of these enigmatic genetic elements. Another example of an extra-chromosomal selfish element is 2 µm plasmids in yeasts that occur at high copy number but are not known to confer a benefit to the host cell. In this issue, Mereshchuk et al. investigate the multicopy yeast plasmid *pSB3* (Mereshchuk et al. 2026) and show that the mechanism of plasmid partitioning and inheritance is conserved across divergent yeast plasmids.

Selfish elements are also found outside of the nucleus. For instance, *Wolbachia* are maternally transmitted endosymbiotic bacteria that infect many arthropod species (reviewed in Porter and Sullivan 2023). *Wolbachia* can manipulate host reproduction to ensure their transmission. One strategy is cytoplasmic incompatibility, where embryos fail to develop when *Wolbachia*-infected males mate with incompatible females. Two papers in this issue explore aspects of cytoplasmic incompatibility systems. Amoros et al. use genomic approaches to study the organization of multicopy genes involved in cytoplasmic incompatibility (*cidA* and *cidB*) in the *Wolbachia* that infects the mosquito *Culex pipien*s, *wPip* (Amoros et al. 2026). They find that *cidA* and *cidB* copies occur in genomic islands and suggest that recurrent structural rearrangements may contribute to the complexity of cytoplasmic incompatibility. Shropshire et al. (2026) calibrate *Wolbachia* genome evolution and reconstruct the evolutionary history of genes involved in cytoplasmic incompatibility (*cif* genes) in closely related *Wolbachia* lineages that infect diverse species. Their work provides new insights into the evolutionary history of cytoplasmic incompatibility systems and timescales of host switching in well-studied model *Wolbachia* clades (Shropshire et al. 2026).

## Synthetic selfish genetic element systems

As illustrated by the articles discussed above, the study of naturally occurring selfish genetic elements has broadened and deepened our understanding of evolutionary processes. Applied evolutionary biologists have in parallel been using the discovered characteristics of these naturally evolved elements as blueprints for developing and inserting novel SSGEs into genomes of mosquitoes and other insect pests. The goal of this work is to spread genetic traits that either suppress a pest population or decrease the ability of the pest to transmit pathogens. Population genetic models are used to predict how specific SSGEs would behave in different pest populations, and recent advances in genomics and genetic engineering techniques enable the production of prototype SSGE strains of malaria-vectoring mosquitoes and agricultural pests. If and when some genetically well-characterized SSGEs are released into the environment, their spread and impact will likely be carefully monitored and could therefore provide general insights about dynamics of less well-understood natural selfish elements. In the meantime, researchers are focusing on investigating potential limitations of current SSGEs while developing ways of improving their efficiency and stability. The studies published in this series provide examples of these types of research efforts.

The malaria parasite, *Plasmodium falciparum*, is well known for its ability to adapt to antimalarial medicines that only have a single active chemical ingredient. Therefore, it would not be surprising if an engineered mosquito with a single genetic trait for inhibiting the parasite did not last long. Thus, researchers have been developing strains of mosquitoes that contain 2 different means of interfering with the parasite (ie effector genes). In this issue, Carballar-Lejarazú et al. have taken this a step further by inserting 4 unique antiparasite traits (Carballar-Lejarazú et al. 2026). In addition to these effector genes, the strains had SSGE properties based on homology-directed repair (HDR) mechanisms. This involves the transgenic construct having sequences on each end that are homologous to a specific site in the insect's genome. This construct must also have a Cas9 gene and guide RNA (gRNA) genes to cut the DNA at that site during the early stages of gametogenesis. This is accomplished by having the Cas9 gene driven by the promoter of one of the target species' gametogenesis-specific genes. Unfortunately, when these promoters are taken out of their natural genomic locations and placed in a construct, their expression can become “leaky” with undesired Cas9 in somatic tissues causing problematic cleavage. To address this issue, Yen et al. develop an integral gene drive where the construct minus a Cas9 promoter is inserted into a location near the natural promoter and where Cas9 expression becomes controlled by that promoter (Yen et al. 2026). This is expected to result in more targeted expression and a lower chance of resistance evolving to the SSGE. This strain requires further modification, but it did drive to high frequencies in caged populations (Yen et al. 2026).

Three papers in this series focus on examining components of SSGEs that could result in failures of releases to spread into natural populations. In initial studies of an SSGE, the engineered strain is released into a caged population of a lab strain to determine if the engineered construct will increase in frequency. These lab strains are genetically fairly homogeneous. In contrast, the wild populations into which the engineered strains are expected to be released are genetically variable. It is acknowledged that genetic variation right at the site targeted by the gRNAs would likely cause failure, so researchers search for sites that are estimated to be fixed or nearly fixed in the species. What has been less investigated is whether differences between the genetic sequences of the homology arms at each end of the transgenic construct and the sequences of the insect that they are meant to match will decrease HDR. Harvey-Samuel et al. directly investigate this issue. They find that a ∼9% difference between the sequences results in a ∼50% decrease in HDR. This is especially significant in the mosquito studied, *Culex quinquefasciatus,* because its HDR rate, even with perfect matching, is low in comparison to other mosquitoes (Harvey-Samuel et al. 2026).

Cas9 alone or with gRNAs can cause double-stranded DNA (dsDNA) breaks at off-target sites, so if an SSGE with these elements was used to suppress the pathogen vectoring capacity of a mosquito population, these genes could plausibly cause accumulation of mutations in that population and decrease its fitness. This could impede that population from spreading the SSGE to other populations. To get a first estimate on what the equilibrium level of mutation accumulation could be, Overton et al. use the yeast *Saccharomyces cerevisiae* as a model system (Overton et al. 2026). They use loss of heterozygosity event rate as a proxy for the dsDNA break rate. While they find that presence of Cas9 alone or with gRNAs did likely elevate the rate of breaks, the increase was small compared with natural variation in mutation rate found among strains of this yeast. While the authors conclude that based on the yeast system the risks “are probably acceptable,” they caution that there are big biological differences between yeasts and multicellular organisms and that further experiments are warranted.

When something goes wrong with a strain engineered with an SSGE, the sleuthing begins. There are many likely culprits and some that are generally overlooked. Sarig et al. develop a transgenic mosquito line that unexpectedly has females unable to blood-feed and are therefore sterile, while males initially appeared normal and fertile but have a reduced life span (Sarig et al. 2026). On closer inspection, they find that both sexes have a distally curved proboscis that impacts feeding. After discounting other hypotheses, the researchers establish that the DsRed marker gene is the cause. Curiously, a second strain with different flanking sequences for the DsRed marker has no noticeable negative traits. How a specific marker gene construct can affect morphology is yet to be explained. The lesson from this study is that biology is too complicated to allow for general assumptions.

In addition to controlling pests or pathogens, selfish drive systems can be used to improve animal and plant breeding so that desired traits are preferentially inherited. For instance, in livestock breeding, individuals of one sex are often culled, and a mechanism to bias the offspring sex ratio could lead to both ethical and financial benefits. In this issue, Bauer et al. use a component of the naturally occurring *t*-haplotype drive system to create an efficient sex ratio distortion system in mice (Bauer et al. 2026). The *t*-haplotype is a natural variant of chromosome 17 in mice that favors its own transmission in *t*/+ males. When the distorter is not present, sperm carrying the *t*-complex-responder (*Tcr*) have low viability. By placing *Tcr* transgene into defined landing sites on the X or Y chromosome, the authors demonstrate efficient sex ratio distortion in mice in favor of either male or female nontransgenic progeny (Bauer et al. 2026).

A final paper in this series exemplifies synergies that can develop between studies of natural and synthetic selfish elements. Mouse *t*-haplotypes are some of the most intensively studied natural selfish elements, yet the many models developed to predict their dynamics in the wild yield results that contrast sharply with empirical measurements. The Thomas research group have been engineering the *t*-haplotype in mice to include a sequence that dominantly abolishes activity of a fertility gene. The goal is environmental release of these constructs to suppress invasive mice on islands, but this brings up many regulatory and ethical issues. To address some of these issues, the group developed complex, spatially explicit, stochastic models (Gierus et al. 2022). Here, Birand et al. (2026) further develop this model structure to address questions about dynamics of *t*-haplotypes in natural populations. In contrast to previous models, Birand et al. (2026) include an array of ecological factors. The results fit with empirical measurements and offer a complex understanding of why some *t*-haplotypes coexist, outcompete each other, and/or occur at low frequencies. The motivation to carefully address an applied problem has resulted in a better understanding of a natural trait.

In summary, selfish elements have captured the attention of biologists since the early days of genetics, and we hope that this outstanding set of publications showcases recent exciting advances as well as motivates further studies in this field. We now know selfish elements take on many forms and are ubiquitous across the tree of life, and we are discovering the mechanisms they use, their genomic features, and the dynamics of their spread through a population. Recent biotechnology advances allow for the development of synthetic drive systems to suppress or alter traits of target species, and with this power comes the responsibility to understand the potential unintentional consequences of these systems. We expect that both natural and synthetic selfish element systems will continue to capture the fascination of geneticists, and we hope that GENETICS and G3 continue to be the premier place to share work on selfish non-Mendelian transmission. We encourage authors to continue to submit their original work to this series, and we also welcome commentaries and perspectives. Accepted articles will be added to the online collection.
